# Time-course microarray analysis for identifying candidate genes involved in obesity-associated pathological changes in the mouse colon

**DOI:** 10.1186/s12263-016-0547-x

**Published:** 2016-11-22

**Authors:** Yun Jung Bae, Sung-Eun Kim, Seong Yeon Hong, Taesun Park, Sang Gyu Lee, Myung-Sook Choi, Mi-Kyung Sung

**Affiliations:** 1 Division of Food Science and Culinary Arts, Shinhan University, Gyeonggi-do, Republic of Korea; 2 Department of Food and Nutrition, Sookmyung Women’s University, 100 Cheongpa-ro 47-gil, Yongsan-gu, Seoul Republic of Korea; 3 Department of Food and Nutrition, Yonsei University, Seoul, Republic of Korea; 4 School of Life Science and Biotechnology, Kyungpook National University, Daegu, Republic of Korea; 5 Department of Food Science and Nutrition, Kyungpook National University, Daegu, Republic of Korea; 6 Food and Nutritional Genomics Research Center, Kyungpook National University, Daegu, Republic of Korea

**Keywords:** Obesity, Colorectal cancer, Time-course microarray analysis, Gene expression, Clustering, Virtual network analysis

## Abstract

**Background:**

Obesity is known to increase the risk of colorectal cancer. However, mechanisms underlying the pathogenesis of obesity-induced colorectal cancer are not completely understood. The purposes of this study were to identify differentially expressed genes in the colon of mice with diet-induced obesity and to select candidate genes as early markers of obesity-associated abnormal cell growth in the colon.

**Methods:**

C57BL/6N mice were fed normal diet (11% fat energy) or high-fat diet (40% fat energy) and were euthanized at different time points. Genome-wide expression profiles of the colon were determined at 2, 4, 8, and 12 weeks. Cluster analysis was performed using expression data of genes showing log_2_ fold change of ≥1 or ≤−1 (twofold change), based on time-dependent expression patterns, followed by virtual network analysis.

**Results:**

High-fat diet-fed mice showed significant increase in body weight and total visceral fat weight over 12 weeks. Time-course microarray analysis showed that 50, 47, 36, and 411 genes were differentially expressed at 2, 4, 8, and 12 weeks, respectively. Ten cluster profiles representing distinguishable patterns of genes differentially expressed over time were determined. Cluster 4, which consisted of genes showing the most significant alterations in expression in response to high-fat diet over 12 weeks, included *Apoa4* (apolipoprotein A-IV), *Ppap2b* (phosphatidic acid phosphatase type 2B), *Cel* (carboxyl ester lipase), and *Clps* (colipase, pancreatic), which interacted strongly with surrounding genes associated with colorectal cancer or obesity.

**Conclusions:**

Our data indicate that *Apoa4*, *Ppap2b*, *Cel*, and *Clps* are candidate early marker genes associated with obesity-related pathological changes in the colon. Genome-wide analyses performed in the present study provide new insights on selecting novel genes that may be associated with the development of diseases of the colon.

**Electronic supplementary material:**

The online version of this article (doi:10.1186/s12263-016-0547-x) contains supplementary material, which is available to authorized users.

## Background

Obesity is a major global health problem that is closely associated with non-communicable diseases with rapidly increasing incidence, including type 2 diabetes, hypertension, cardiovascular diseases, and some cancers [1]. Excess energy intake contributes to abnormal intermediate conditions such as hyperinsulinemia, hyperglycemia, and dyslipidemia, leading to the development of obesity-related metabolic complications [2].

Epidemiological evidence indicates that excess body fat is associated with an increased risk of colorectal cancer (CRC) [3]. The risk of CRC increases by 7% with an increase in body mass index (BMI) by 2% [4]. Experimental studies also indicate that diet-induced obesity causes pathological changes in the colon. The number of polyps is significantly higher, and the areas of hyperplasia in the colonic mucosa and inflammatory foci throughout the gastrointestinal tract are broader in high-fat diet (HFD)-fed mice than in control mice [5]. Mice fed HFD for two third of their life span and not treated with carcinogenic chemicals show substantially higher incidence and multiplicity of colon tumor than mice fed a control diet [6]. Increased circulating concentrations of insulin and leptin are linked to abnormal hyperproliferation of colorectal tissue and inflammation possibly by controlling transcription factors involved in the expression of cell growth-regulating molecules [7–12]. Whole-colon proteomic analyses of wild-type and leptin-deficient *ob/ob* mice suggest that 40 differently expressed proteins are associated with obesity-related pathological changes in the colon [13]. However, to our knowledge, no study has identified candidate molecules involved in obesity-associated pathological changes in the colon of HFD-fed mice. Moreover, limited information is available on mechanisms underlying the pathophysiological changes in the colon tissue of obese animals.

Interactions between nutritional factors and cellular events in the biological system are extremely complicated. Traditional nutrition research design involving one or two molecular targets often cannot explain phenotypic changes induced by missing responses of other important targets to nutritional stimuli. Recent developments in genome-wide analyses have been used to identify biomarkers that respond to nutritional intervention such as HFD. Several studies indicate that diet-induced obesity changes gene expression patterns in various tissues. Expression of key adipose transcription factors that regulate adipogenesis and insulin sensitivity, including leptin, resistin, uncoupling protein-2, tumor necrosis factor-alpha (TNF-α), CCAAT/enhancer-binding protein α, peroxisome proliferator-activated receptor, sterol regulatory element-binding transcription factor 1, and hydroxysteroid 11-beta dehydrogenase 1, is changed in the gonadal fat tissue of HFD-fed animals [14, 15]. HFD also alters the expression of interferon-gamma, interleukin-4, interleukin-10, interleukin-12, and TNF-α in the liver tissue [16]. Despite a strong association between obesity and pathophysiological changes in the small intestine and colon that lead to the development of ulcerative colitis, irritable bowel syndrome (IBD), and CRC, only few studies have examined the association between diet-induced obesity and gene expression pattern of the intestinal tissue [17, 18]. A recent study reported substantial changes in lipid metabolism-related gene expression in the small intestine of animals fed long-chain fatty acids of marine origin [19]. Our present study is the first to report global transcriptional changes at different time points during the development of diet-induced obesity in the colon of HFD-fed animals. In addition, we performed bioinformatics analyses to identify candidate early marker genes that might be involved in obesity-related pathological events such as CRC and IBD.

## Methods

### Animals

This study was performed in accordance with the Guide for the Care and Use of Laboratory Animals developed by the Institute of Laboratory Animal Resources of the National Research Council [20] and was approved by the Institutional Animal Care and Use Committee of Yonsei University in Seoul, Republic of Korea (Permit No.: 2010-0039). Eighty 5-week-old male C57BL/6N mice (Orient, Gyeonggi-do, Korea) were housed in a temperature (21 ± 2 °C)- and humidity (50 ± 5%)-controlled room with a 12-h light/12-h dark cycle. The mice were fed a commercial diet (Purina, St. Louis, MO, USA) for 1 week and were randomly assigned to receive normal diet (ND, *n* = 40) and HFD (*n* = 40). HFD contained 200 g fat/kg (170 g lard plus 30 g corn oil) and 1% cholesterol by weight. It was formulated to provide 40% of the total energy from fat by replacing carbohydrates with lard and corn oil; however, it contained the same amount of vitamins and minerals per kilocalorie as those in the ND. Compositions of the experimental diets are presented in Additional file 1: Table S1. The mice were fed the experimental diets and water *ad libitum.* Food intake of the mice was recorded daily, and their body weights were measured every 3 days. Ten mice per group were sacrificed at 2, 4, 8, and 12 weeks of feeding the experimental diets by fasting them overnight and by anesthetizing them with diethyl ether. Their colons were laid flat on a glass plate, and the colonic mucosa was scraped using a glass slide. The colon samples were stored at −80 °C until their use.

### Time-course microarray analysis

**Fig. 1:**
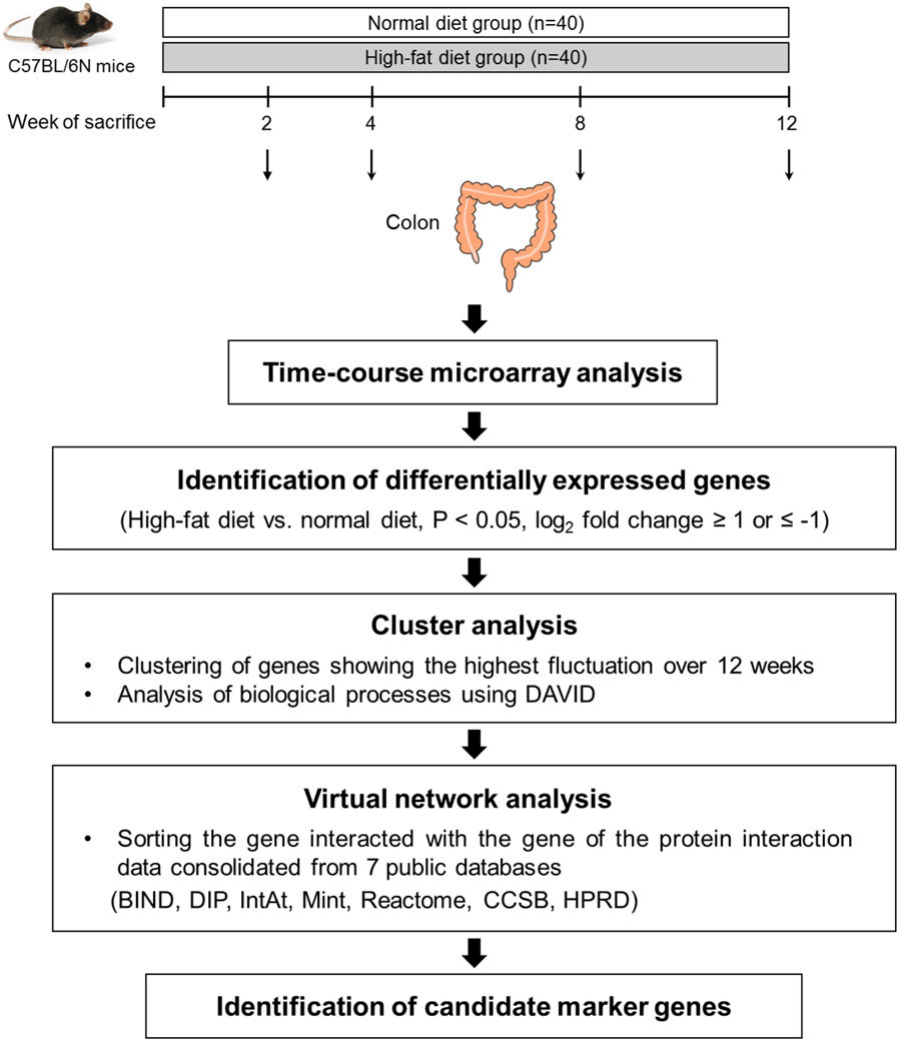
Schematic flow chart of the study design representing time-course microarray analyses (2, 4, 8, and 12 weeks) of the colon tissue of C57BL/6N mice fed normal or high-fat diet

Total RNA was isolated from the colon tissue of each mouse, using TRIzol (Invitrogen Life Technologies, Carlsbad, CA, USA), and was purified using RNeasy column (Qiagen, Valencia, CA, USA), according to the manufacturer’s protocols. RNA purity and integrity were evaluated by denaturing gel electrophoresis, OD_260_/OD_280_ ratio, and analyzed on the Agilent 2100 Bioanalyzer (Agilent Technologies, Palo Alto, CA, USA). The RNA Integrity Number (RIN) score was generated on the Agilent software, and the average RIN score of all samples used for microarray analysis was 8.5 ± 0.9 (mean ± SD). To reduce individual variability in gene expression, identical amounts of total colonic RNA were pooled from 10 mice in each experimental group and a pooled RNA sample representing the ND and HFD group at 2, 4, 8, and 12 weeks was subjected to microarray experiment as described previously [21]. Total RNA was amplified and purified using the Illumina® TotalPrepTM-96 RNA Amplification Kit (Ambion, Austin, TX, USA) to produce biotinylated complementary RNA (cRNA), according to the manufacturer’s instructions. The cRNA obtained was quantified using an ND-1000 Spectrophotometer (NanoDrop, Wilmington, DE, USA). The biotinylated cRNA was hybridized onto the Illumina Mouse WG-6 v2.0 Expression BeadChip (Illumina, Inc., San Diego, CA, USA) containing 45,281 probes representing 30,584 genes. After washing and staining, the BeadChip was scanned with the Illumina Bead Array Reader Confocal Scanner according to the manufacturer’s instructions. Raw data were exported and analyzed using BeadStudio v3.1.3 (Gene Expression Module v3.3.8; Illumina). All the data analyses and visualization of differentially expressed genes were conducted using ArrayAssist® (Stratagene, La Jolla, CA, USA). Values are expressed as log_2_ fold change and were obtained by comparing the gene expression profiles of HFD-fed mice with those of ND-fed mice. Genes showing log_2_ fold change of ≥1 or ≤−1 (fold change of ≥2 or ≤−2) were selected, and functional analysis was performed using PANTHER database system (www.patherdb.org). Clustering analysis was performed using genes showing similar expression trends over time. MultiExperiment Viewer program was used to evaluate K-means algorism [22]. A gene cluster showing the highest fluctuation over time was selected, and biological processes associated with these HFD-responsive genes over time were analyzed using Database for Annotation, Visualization and Integrated Discovery (DAVID, https://david.ncifcrf.gov/) [23]. Virtual interaction network-targeted genes in the selected cluster were determined using Michigan Molecular Interactions software [24, 25]. In this network, genes that interacted with genes in the protein interaction data consolidated from seven public databases (Biomolecular Interaction Network Database [BIND], Database of Interacting Proteins [DIP], IntAct molecular interaction database [IntAct], Molecular INTeraction database [Mint], Reactome, CCSB Interactome Database [CCSB], and Human Protein Reference Database [HPRD]) were sorted [26–28] (Fig. 1).

### Real-time quantitative polymerase chain reaction analysis

Real-time quantitative polymerase chain reaction (qPCR) was conducted to validate microarray data of several differentially expressed genes that were selected based on the clustering and network analyses and that were associated with the biological function of interest, including CRC and obesity. Template RNA isolated from the colon tissue was reverse transcribed using Superscript™ II RT-PCR System (Invitrogen, Karlsruhe, Germany), according to the manufacturer’s instructions, for performing dT 20-primed complementary DNA (cDNA) synthesis. Next, real-time qPCR was performed using an ABI PRISM 7900HT Sequence Detection System (Applied Biosystems, Foster City, CA, USA) in 384-well microtiter plates containing a final reaction volume of 10 μl. Four primer/TaqMan probe combinations were designed based on the following sequences obtained from an NCBI public database: *Apoa4*, Mm00431814_m1; *Cel*, Mm00486975_m1; *Clps*, Mm00517960_m1; and *Ppap2b*, Mm00504516_m1. Amplifications were performed using the following protocol: initial template denaturation at 95 °C for 10 min, followed by 40 cycles at 95 °C for 15 s and 60 °C for 1 min. All the samples were amplified in triplicate, and data were analyzed using Sequence Detector software (Applied Biosystems).

### Statistical analysis

Differences among mice in the two dietary groups were analyzed by Student’s *t* test, with SAS 9.4 (SAS Institute, Inc., Cary, NC, USA). Results were considered statistically significant if two-tailed *P* values were <0.05.

## Results

### Time-course of changes in body weight, visceral fat pad weight, and food efficiency ratio during the development of diet-induced obesity

**Fig. 2:**
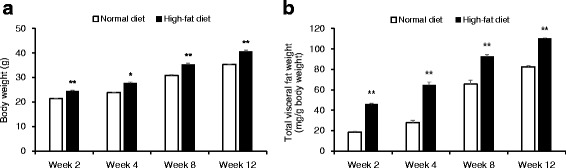
Time-course changes in body weight and total visceral fat weight during the development of diet-induced obesity. **a** Body weight. **b** Total visceral fat weight. Data are presented as mean ± SEM (*n* = 10/diet group). *P < 0.01 and **P < 0.001 indicate significant difference compared with mice in the normal diet group

C57BL/6N mice fed HFD for 2 weeks gained significantly more weight than mice fed ND (*P* < 0.001; Fig. 2a). At the end of 12 weeks, HFD-fed mice gained 22.3 g weight compared with ND-fed mice that gained 15.3 g weight (*P* < 0.001). Total visceral fat weight of HFD-fed mice was higher than that of ND-fed mice at as early as 2 weeks of the experiment (*P* < 0.001; Fig. 2b). Food efficiency ratio also increased significantly for HFD-fed mice at all the time points compared with that for ND-fed mice (*P* < 0.001; Additional file 2: Table S2).

### Time-course of transcriptional changes in the colon tissue during the development of diet-induced obesity

Two-dimensional hierarchical clustering showed different gene expression patterns at different time points in HFD- and ND-fed C57BL/6N mice (Additional file 3: Figure S1). The number of HFD-responsive genes at different time points in the colon of C57BL/6N mice is presented in Table 1. We found that 41, 35, 1, and 33 genes were upregulated and 9, 12, 35, and 378 genes were downregulated at 2, 4, 8, and 12 weeks, respectively, in response to HFD (Table 1). Genes affected by HFD at different time points in the colon tissue of C57BL/6N mice are listed in Additional file 4: Table S3.

**Table 1:** The number of differentially expressed genes affected by the high-fat diet at different time points in the colon tissue of C57BL/6N mice

Weeks	Upregulated	Downregulated	Total
2	41	9	50
4	35	12	47
8	1	35	36
12	33	378	411

Differentially expressed genes indicate genes showing a log_2_ fold change of ≥1 or ≤−1 (fold change of ≥2 or ≤−2) based on the comparison between high-fat diet- and normal diet-fed mice at each time point (*P* < 0.05)

Functional analysis was performed using PANTHER classification system to identify biological processes associated with HFD-responsive genes in the colon tissue of mice. The biological processes associated with HFD-responsive genes in the colon tissue of mice are presented in Table 2. At week 12, HFD affected several biological processes, including immunity and defense; nucleoside, nucleotide, and nucleic acid metabolism; signal transduction; and cell cycle (Table 2). Biological processes associated with HFD-responsive genes at different time points in the colon tissue of C57BL/6N mice are listed in Additional file 5: Table S4.

**Table 2:** The biological processes associated with high-fat diet-responsive genes at week 12 in the colon tissue of C57BL/6N mice

Biological process	No. of total probes	No. of genes		*P* value	FDR
		Up	Down		
Immunity and defense	2634	0	28	<0.0001	0.001
Nucleoside, nucleotide, and nucleic acid metabolism	5188	4	55	<0.001	0.005
Signal transduction	7336	1	60	0.014	0.131
Cell cycle	1692	1	16	0.039	0.262
Developmental processes	3927	2	37	0.047	0.262

*FDR* false discovery rate using a Benjamini and Hochberg multiple testing correction, *P value* modified Fisher’s exact *P* value calculated by PANTHER database system

We also identified HFD-responsive genes showing log_2_ fold change of ≥1 or ≤−1 (corresponding to a fold change of ≥2 or ≤−2) at multiple time points (>3 times) over 12 weeks (Table *3*). Most HFD-responsive genes were associated with digestive enzymes such as trypsin, carboxypeptidase, and amylase. Overall, these genes were upregulated at weeks 2 and 4 and were downregulated at week 12 in HFD-fed mice compared with those ND-fed mice (Table *3*). *Cfd*, complement factor D (adipsin), was downregulated at weeks 4, 8, and 12 in HFD-fed mice. Adipsin is suggested to activate an alternative complement pathway for inducing natural defense against infectious agents and red cell lysis and to regulate systemic energy balance [29, 30]. A previous study reported that adipsin expression in the small intestine is a potential marker of changes in normal intestinal epithelial differentiation [31]. *Pla2g1b*, pancreatic phospholipase A2, was upregulated at weeks 2 and 4 and was downregulated at week 12 in HFD-fed mice. Pancreatic phospholipase A2 catalyzes the release of fatty acids from dietary phospholipids. Diet is the ultimate source of arachidonic acid present in cellular phospholipids, which serve as precursors of eicosanoid signaling molecules and are involved in inflammation, cell proliferation, and colorectal carcinogenesis. Arachidonic acid is metabolized by PTGS (COX)/LOX pathway to prostaglandins and leukotrienes, which are associated with carcinogenesis, specifically of colonic carcinogenesis [32, 33].

**Table 3:** Genes expressed differentially in response to high-fat diet at multiple time points in the colon tissue of C57BL/6N mice

Symbol	Accession	Definition	Log_2_ fold change			
			Week 2	Week 4	Week 8	Week 12
*AI747448*	NM_001033199.2	Expressed sequence AI747448 (AI747448), mRNA.	1.14	−2.62	−1.88	
*Amy2*	NM_009669.1	Amylase 2, pancreatic (Amy2), mRNA.	3.92	1.91		−4.93
*Apoa4*	NM_007468.2	Apolipoprotein A-IV (Apoa4), mRNA.	1.06		−1.20	−1.13
*Cel*	NM_009885.1	Carboxyl ester lipase (Cel), mRNA.	3.99	1.26		−3.88
*Cfd*	NM_013459.1	Complement factor D (adipsin) (Cfd), mRNA.		−1.93	−1.17	−1.19
*Cpa2*	NM_001024698.2	Carboxypeptidase A2, pancreatic (Cpa2), mRNA.	2.95	1.01		−1.91
*Cpb1*	NM_029706.1	Carboxypeptidase B1 (tissue) (Cpb1), mRNA.	4.35	1.31		−4.14
*Ctrb1*	NM_025583.1	Chymotrypsinogen B1 (Ctrb1), mRNA.	4.08	1.64		−2.84
*Ctrl*	NM_023182.2	Chymotrypsin-like (Ctrl), mRNA.	4.04	1.01		−3.52
*Ela2a*	NM_007919.2	Elastase 2A (Ela2a), mRNA.	5.52	1.08		−6.00
*Ela3b*	NM_026419.1		4.62	1.22		−4.12
*LOC100040233*	XM_001474605.1	Predicted: similar to trypsinogen 15 (LOC100040233), mRNA.	3.23	1.27		−2.06
*Pla2g1b*	NM_011107.1	Phospholipase A2, group IB, pancreas (Pla2g1b), mRNA.	3.79	1.34		−2.86
*Rnase1*	NM_011271.2	Ribonuclease, RNase A family, 1 (pancreatic) (Rnase1), mRNA.	4.27	1.29		−3.58
*Try10*	NM_001038996.1	Trypsin 10 (Try10), mRNA.	3.73	1.55		−2.84
*Try4*	NM_011646.5	Trypsin 4 (Try4), mRNA.	4.72	1.34		−3.67

Log_2_ fold change of ≥1 or≤−1 corresponds to a fold change of ≥2 or ≤−2, respectively, based on a comparison between high**-**fat diet**-** and normal diet**-**fed mice at each time point (*P* < 0.05)

### Cluster and network analyses for identifying candidate early marker genes associated with diet-induced obesity

**Fig. 3:**
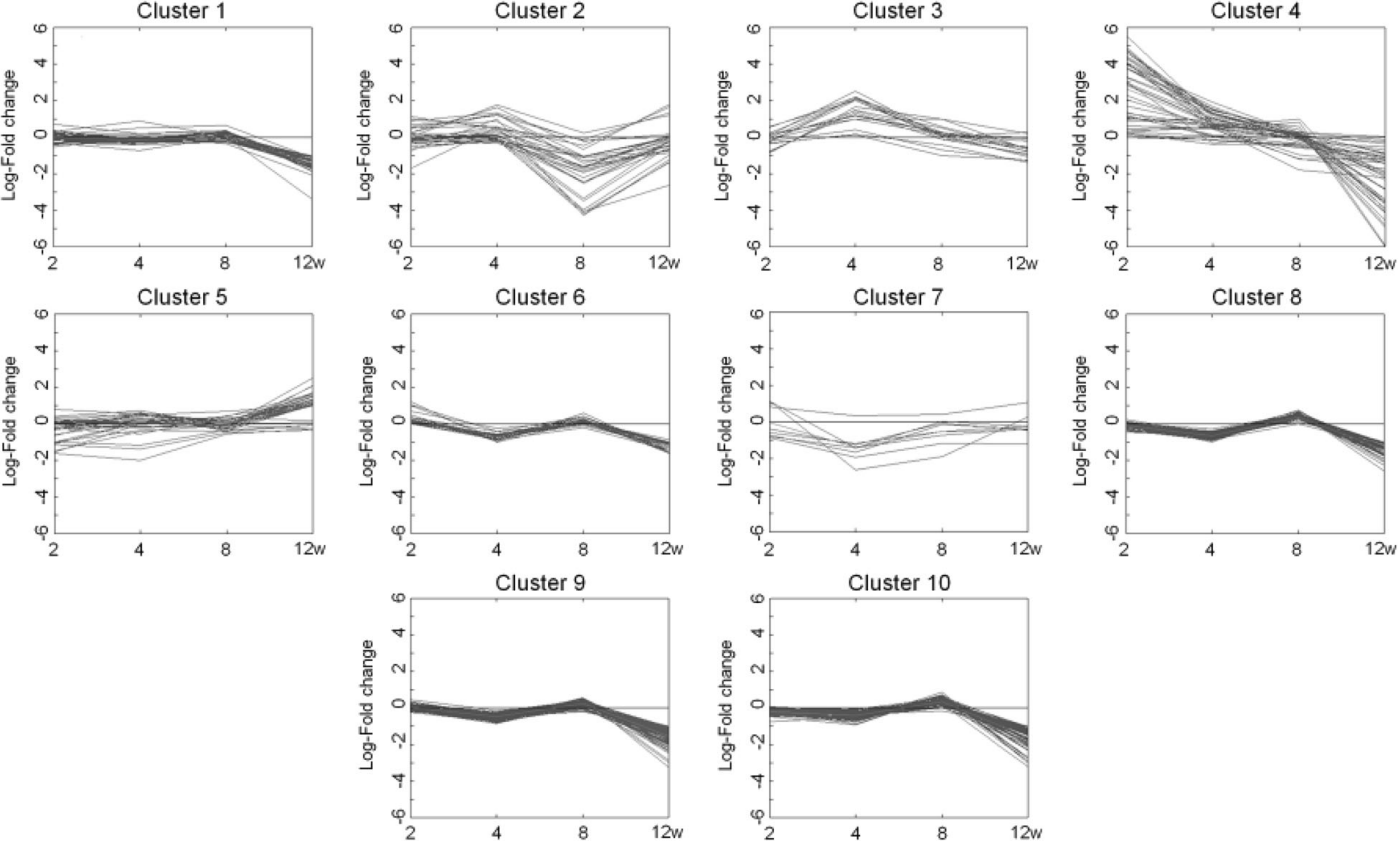
Clustering analysis of genes expressed differentially in response to high-fat diet over 12 weeks in the colon tissue of C57BL/6N mice

**Fig. 4:**
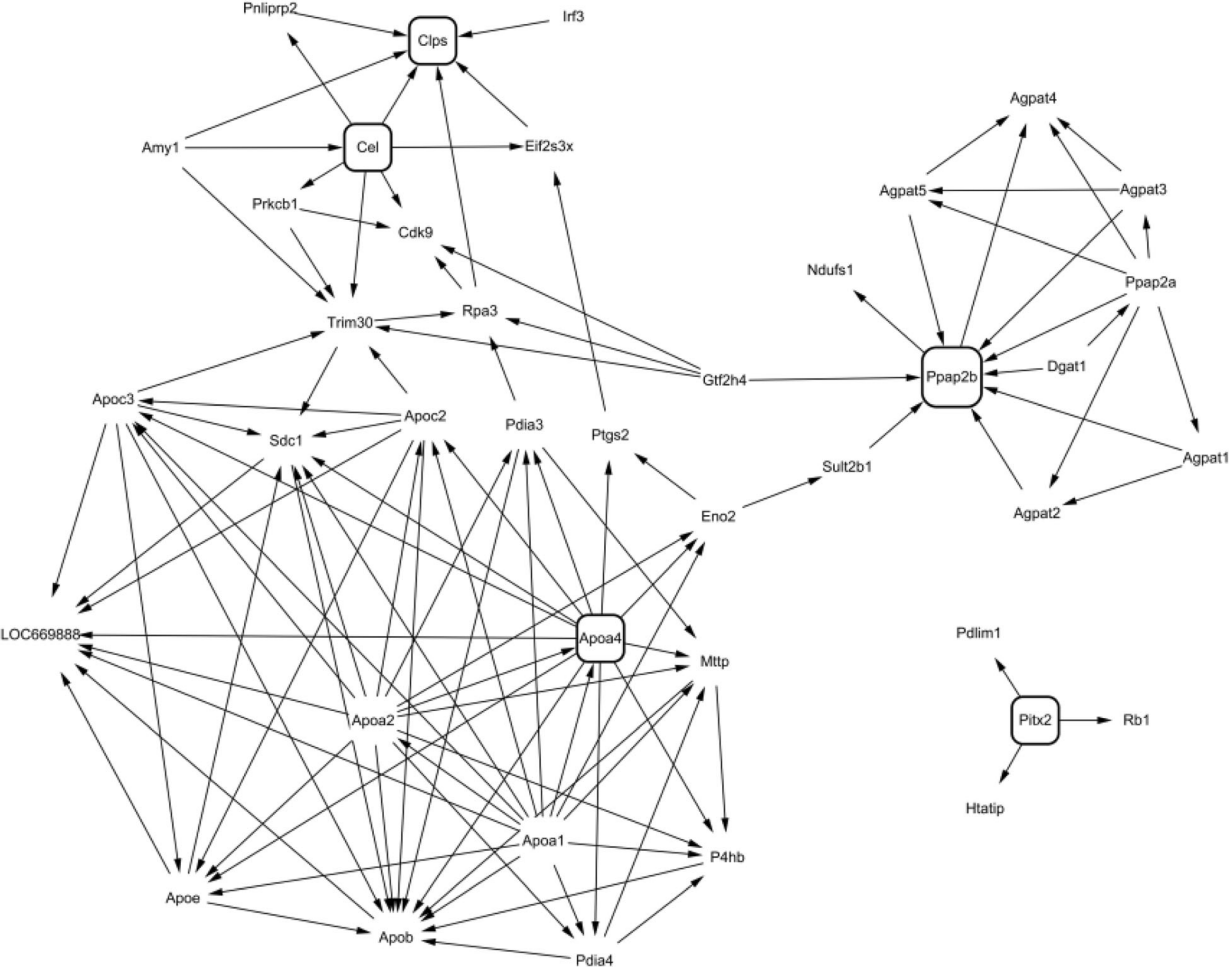
Virtual network analysis of the selected genes in cluster 4

**Fig. 5:**
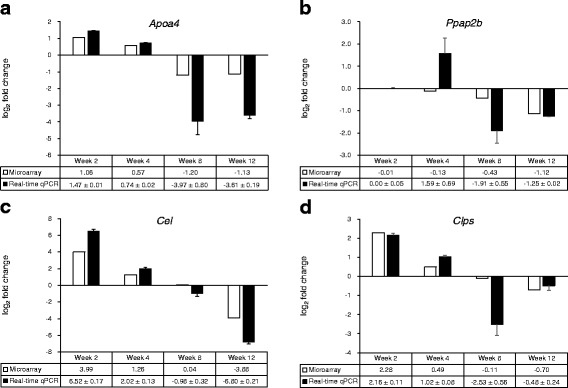
Comparison of log_2_ fold changes in gene expression detected by performing microarray and quantitative reverse transcription-PCR analyses. Microarray data are presented as log_2_-transformed mean fold changes (high-fat diet vs. normal diet) in gene expression at each time point. The real-time qPCR data are shown as log_2_-transformed mean fold changes ± SEM of the RNA samples pooled from 10 mice per group (*n* = 3). **a** *Apoa4*, apolipoprotein A-IV. **b** *Ppap2b*, phosphatidic acid phosphatase type 2B. **c** *Cel*, carboxyl ester lipase. **d** *Clps*, colipase, pancreatic

We next selected a cluster of HFD-responsive genes showing the highest fluctuation over time. Ten separate cluster profiles showing distinguishable patterns of genes expressed differentially over time were determined (Fig. 3). The number of genes in each cluster was as follows: cluster 1, 45 genes; cluster 2, 32 genes; cluster 3, 17 genes; cluster 4, 44 genes; cluster 5, 35 genes; cluster 6, 24 genes; cluster 7, 8 genes; cluster 8, 78 genes; cluster 9, 103 genes; and cluster 10, 76 genes. Virtual network analysis was performed for genes in cluster 4 that showed the most significant alterations in response to HFD over 12 weeks. The genes in cluster 4 are listed in Table 4. Gene ontology (GO) biological pathway analysis showed that genes in cluster 4 were involved in proteolysis, lipid catabolic process, digestion, defense response, and acute-phase response (Table 5). Results of the virtual network analysis showed that *Apoa4* (apolipoprotein A-IV), *Ppap2b* (phosphatidic acid phosphatase type 2B), *Cel* (carboxyl ester lipase), and *Clps* (colipase, pancreatic) strongly interacted with surrounding genes (Fig. 4). Previous studies have reported that these core genes are involved in pathological changes associated with CRC or obesity [34–36]. Results of microarray-based analysis of the expression of these genes were confirmed by performing real-time qPCR at each time point. Overall, the changes in the transcription profiles of *Apoa4*, *Ppap2b*, *Cel*, and *Clps* determined by real-time qPCR were consistent with the results of microarray analysis (Fig. 5). The direction of change between the two analyses was consistent for the significantly regulated genes except *Ppap2b* at week 4 (log_2_ fold change −0.13) and *Cel* at week 8 (log_2_ fold change 0.04).

**Table 4:** Genes in cluster 4

Symbol	Accession	Definition	Log_2_ fold change			
			Week 2	Week 4	Week 8	Week 12
*1810033M07Rik*	NM_026983.1	RIKEN cDNA 1810033M07 gene (1810033M07Rik), mRNA.	1.30	0.67	0.22	−0.90
*1810049H19Rik*	NM_001003405.1	RIKEN cDNA 1810049H19 gene (1810049H19Rik), mRNA.	4.62	1.44	0.41	−3.66
*2900060B14Rik*			1.38	0.07	0.11	−0.36
*Amy2*	NM_009669.1	Amylase 2, pancreatic (Amy2), mRNA.	3.92	1.91	0.22	−4.93
*Amy2-2*	NM_001042711.2	Amylase 2-2, pancreatic (Amy2-2), mRNA.	4.02	0.44	0.82	−5.93
*Apoa4*	NM_007468.2	Apolipoprotein A-IV (Apoa4), mRNA.	1.06	0.57	−1.20	−1.13
*Cel*	NM_009885.1	Carboxyl ester lipase (Cel), mRNA.	3.99	1.26	0.04	−3.88
*Clps*	NM_025469.2	Colipase, pancreatic (Clps), mRNA.	2.28	0.49	−0.11	−0.70
*Cpa1*	XM_284174.1		4.89	0.78	0.15	−4.54
*Cpa2*	NM_001024698.2	Carboxypeptidase A2, pancreatic (Cpa2), mRNA.	2.95	1.01	0.18	−1.91
*Cpb1*	NM_029706.1	Carboxypeptidase B1 (tissue) (Cpb1), mRNA.	4.35	1.31	0.02	−4.14
*Ctrb1*	NM_025583.1	Chymotrypsinogen B1 (Ctrb1), mRNA.	4.08	1.64	0.04	−2.84
*Ctrl*	NM_023182.2	Chymotrypsin-like (Ctrl), mRNA.	4.04	1.01	−0.01	−3.52
*Cuzd1*	NM_008411.3	CUB and zona pellucida-like domains 1 (Cuzd1), mRNA.	1.70	0.72	−0.04	−1.25
*Cygb*	NM_030206.1	Cytoglobin (Cygb), mRNA.	0.12	−0.15	−0.51	−1.28
*EG386551*	NM_001003664.1	Predicted gene, EG386551 (EG386551), mRNA.	4.43	1.48	0.28	−3.41
*EG436523*	NM_001038997.1	Predicted gene, EG436523 (EG436523), mRNA.	3.28	1.50	0.15	−1.97
*Ela1*	NM_033612.1	Elastase 1, pancreatic (Ela1), mRNA.	2.04	0.61	0.21	−0.87
*Ela2a*	NM_007919.2	Elastase 2A (Ela2a), mRNA.	5.52	1.08	0.60	−6.00
*Ela3b*	NM_026419.1		4.62	1.22	0.04	−4.12
*Golga2*	NM_133852.2	Golgi autoantigen, golgin subfamily a, 2 (Golga2), transcript variant 1, mRNA.	1.21	0.03	−0.59	−0.91
*Gp2*	NM_025989.1	Glycoprotein 2 (zymogen granule membrane) (Gp2), mRNA.	3.78	0.91	−0.15	−2.07
*Hamp2*	NM_183257.1	Hepcidin antimicrobial peptide 2 (Hamp2), mRNA.	1.00	1.00	−0.29	−0.53
*LOC100040233*	XM_001474605.1	Predicted: similar to trypsinogen 15 (LOC100040233), mRNA.	3.23	1.27	0.04	−2.06
*LOC100047162*	XM_001477552.1	Predicted: similar to immunoglobulin kappa-chain (LOC100047162), mRNA.	0.62	0.51	−0.09	−1.40
*LOC272683*	XM_195289.1		0.32	0.07	−0.36	−1.03
*LOC636875*	XM_992953.1	Predicted: similar to Ig kappa chain V-V region L7 precursor (LOC636875), mRNA.	3.00	−0.33	0.16	−1.15
*LOC636944*	XM_912513.1	Predicted: similar to Ig kappa chain V-V region K2 precursor (LOC636944), mRNA.	1.04	0.69	0.24	−0.14
*LOC669053*	XM_972773.1	Predicted: similar to Ig kappa chain V-V region MPC11 precursor (LOC669053), mRNA.	0.30	0.62	−0.96	−1.42
*LOC674114*	XM_975388.1	Predicted: similar to Ig heavy chain V region 5-84 precursor (LOC674114), mRNA.	1.15	0.14	0.26	0.04
*Pdia2*	NM_001081070.1	Protein disulfide isomerase associated 2 (Pdia2), mRNA.	2.89	0.81	0.09	−1.97
*Pitx2*	NM_001042502.1	Paired-like homeodomain transcription factor 2 (Pitx2), transcript variant 3, mRNA.	0.48	0.56	−1.81	−2.20
*Pitx2*	NM_011098.3	Paired-like homeodomain transcription factor 2 (Pitx2), transcript variant 2, mRNA.	0.40	0.45	−1.17	−1.72
*Pla2g1b*	NM_011107.1	Phospholipase A2, group IB, pancreas (Pla2g1b), mRNA.	3.79	1.34	−0.02	−2.86
*Pnlip*	NM_026925.3	Pancreatic lipase (Pnlip), mRNA.	4.78	0.75	0.20	−4.79
*Pnliprp1*	NM_018874.2	Pancreatic lipase related protein 1 (Pnliprp1), mRNA.	4.79	0.58	1.00	−4.00
*Ppap2b*	NM_080555.2	Phosphatidic acid phosphatase type 2B (Ppap2b), mRNA.	−0.01	−0.13	−0.43	−1.12
*Prss3*	NM_011645.1	Protease, serine, 3 (Prss3), mRNA.	0.87	1.02	0.06	−0.25
*Reg1*	NM_009042.1	Regenerating islet-derived 1 (Reg1), mRNA.	4.03	0.59	−0.02	−3.41
*Rnase1*	NM_011271.2	Ribonuclease, RNase A family, 1 (pancreatic) (Rnase1), mRNA.	4.27	1.29	0.22	−3.58
*Saa3*	NM_011315.3	Serum amyloid A 3 (Saa3), mRNA.	0.24	−0.16	−0.46	−1.40
*Serpina3n*	NM_009252.2	Serine (or cysteine) peptidase inhibitor, clade A, member 3N (Serpina3n), mRNA.	0.19	−0.38	−0.35	−1.02
*Tff2*	NM_009363.3	Trefoil factor 2 (spasmolytic protein 1) (Tff2), mRNA.	2.84	0.81	0.15	−2.18
*Try10*	NM_001038996.1	Trypsin 10 (Try10), mRNA.	3.73	1.55	0.09	−2.84
*Try4*	NM_011646.5	Trypsin 4 (Try4), mRNA.	4.72	1.34	0.15	−3.67

Log_2_ fold change of ≥1 or ≤−1 corresponds to a fold change of ≥2 or ≤−2, respectively, based on a comparison between high**-**fat diet**-** and normal diet**-**fed mice at each time point (*P* < 0.05)

**Table 5:** Gene ontology biological pathway analysis of genes in cluster 4

GO term	Pop hits	No. of genes	Genes	*P* value	FDR
Proteolysis	1034	15	*Prss3, Ela3b, Ctrb1, Cpa2, Try10, Ela2a, Ela1, EG436523, EG386551, 1810049H19Rik, LOC100040233, Ctrl, Try4, Cuzd1, Cpb1*	<0.0001	<0.0001
Lipid catabolic process	134	5	*Clps, Pla2g1b, Cel, Pnlip, Pnliprp1*	<0.0001	<0.01
Digestion	26	3	*Clps, Ctrb1, Pnlip*	<0.01	0.059
Defense response	448	4	*Saa3, Apoa4, Hamp2, Serpina3n*	0.053	0.890
Acute-phase response	30	2	*Saa3, Serpina3n*	0.056	0.846

*GO* gene ontology, *FDR* false discovery rate using a Benjamini and Hochberg multiple testing correction, *Pop Hits* number of genes for a particular GO term, *P value* modified Fisher’s exact *P* value calculated by DAVID software

## Discussion

In the present study, we determined global transcriptional changes at different time points during the development of diet-induced obesity in the colon of mice. We also performed bioinformatics analyses to identify candidate genes that could be used as early markers of obesity-related pathological events. Diet-induced obesity is associated with many chronic diseases, including CRC and IBD. Epidemiological studies have reported a significant association between BMI and colon cancer (HR, 1.05; 95% CI, 1.02–1.09) [37]. Obese C57BL/6 mice develop colonic epithelial hyperplasia, and the risk of colon cancer increases by 42% after long-term (18 months) western-style diet feeding [38, 39]. A recent study indicated that HFD increased the number of polyps in the colon and the area of hyperplasia in the mucous membrane tissue of the colon [5]. We previously observed that HFD-fed mice (45% total calories from fat) developed two-times more number of colonic tumors than ND-fed mice possibly because of adipokine-mediated signaling of phosphatidylinositol 3-kinase/Akt pathway [40]. However, limited information is available on mechanisms underlying the associations between obesity and pathophysiological changes in the colon.

In the present study, genes showing differential expression in response to HFD were subjected to clustering and networking analyses. Clustering algorithms are frequently used to group genes with similar expression profiles [41]. This facilitates the visualization of coexpressed genes and allows the identification of genes that concurrently respond to stimuli. We clustered genes that were expressed differentially over time into 10 patterns. Of the 10 clusters, cluster 4 included genes that were the most responsive to HFD. Many of these genes were upregulated after the initiation of HFD and were downregulated gradually as the mice became obese. We postulated that these genes could be used as early markers of the initiation of metabolic changes in the colon.

We found that genes in cluster 4 were involved in proteolysis, lipid catabolic process, digestion, defense response, and acute-phase response. These results indicate that HFD upregulated the expression of genes involved in lipid catabolism and that these genes were downregulated over time possibly due to interactions with other compensatory and/or adaptive mechanisms. Extracellular proteolysis is critical for tumor growth [42]. Trypsin activates protease-activated receptor-2 (PAR-2) and increases COX-2 expression through PAR-2 in Caco-2 cells [43]. These proteolytic activities may promote tumor cell growth and invasion, suggesting that HFD increases the risk of tumor development by facilitating proteolytic activity. Oxidative stress and gene–environment interactions play a significant role in the development of colon cancer [44]. Oxidative stress results from an imbalance in the production of reactive oxygen species and cellular antioxidant defense system. In the present study, genes associated with defense response tended to be downregulated over time during HFD administration and before colon cancer initiation. This result suggests that continuous HFD administration affects defense mechanisms, which in turn may increase the risk of CRC.

For further analysis, genes in cluster 4 were subjected to network analysis by using BIND, DIP, IntAct, Mint, Reactome, CCSB, and HPRD protein–protein interaction databases. Among the genes in cluster 4, four genes showing the most significant relationship with surrounding genes were selected and their expression was verified. Previous studies indicate that these four genes are associated with pathological changes in the colon or with obesity. APOA4 is an intestinally and cerebrally synthesized antiatherogenic plasma apolipoprotein that functions as a satiety factor and anti-inflammatory protein. Intestinal APOA4 synthesis is stimulated by fat intake and is attenuated by intravenous leptin infusion, indicating a close association between fat and energy intake [45]. *Apoa4* expression is altered along with that of other genes involved in epithelial junctional integrity in the intestinal mucosa of patients with IBD [46]. APOA4 stabilizes adherent junctions by interacting with α-catenin and may be involved in the maintenance of junctional integrity. Epithelial tight junctions form a barrier to prevent the movement of pathogens, toxins, and allergens from the intestinal lumen into the tissue, and disruption of these tight junctions may play an important role in the pathogenesis of gastrointestinal diseases [47, 48].

Lipid phosphate phosphatase 3 (LPP3) encoded by *Ppap2b* is an integral membrane glycoprotein that catalyzes the dephosphorylation of several bioactive lipid mediators, including lysophosphatidic acid, sphingosine 1-phosphate, and phosphatidic acid. Moreover, LPP3 functions as a cell-associated integrin ligand [49, 50]. A recent study reported that LPP3 does not promote tumor formation but amplifies β-catenin signaling and cyclin-D1 activity to potentiate the growth of SW480 colon carcinoma [51]. Aberrant activation of PI3K/Akt/mTOR and MAPK/ERK pathways may induce colon tumor growth and progression by increasing β-catenin and cyclin-D1 expression [52, 53].

Carboxyl ester lipase (CEL) encoded by *Cel* is a 74-kDa lipolytic enzyme that hydrolyzes cholesteryl esters, triacylglycerol, phospholipids, and lysophospholipids [54, 55]. This enzyme is synthesized in acinar cells of the pancreas and is stored in zymogen granules. Upon food ingestion, CEL is released into the intestinal lumen where it constitutes 1–5% of total proteins in the pancreatic juice [56]. CEL plays a significant role in catalyzing the absorption of cholesteryl esters from the intestinal lumen and in promoting the formation of large chylomicron [57, 58]. A recent study reported that *Cel*-knockout mice developed a mild diabetic phenotype after the administration of 60% HFD [59]. Since insulin resistance is a risk factor of colon cancer, differential expression of *Cel* in obese animals may be responsible for the association of obesity with the pathophysiological changes in the colon.

*Clps* encodes colipase that is secreted from the exocrine pancreas into the gastrointestinal tract [60]. Colipase may interact with pancreatic triglyceride lipase to facilitate the digestion of dietary fats. HFD-fed *Clps* ^*−/−*^ mice develop hyperphagia, and procolipase performs essential functions by regulating body weight set point [61]. Also, *Clps* genetic variability is associated with insulin secretory function in non-diabetic humans, suggesting that *Clps* is a novel candidate gene associated with the development of type 2 diabetes [36]. Regulation of insulin secretion is important for metabolic homeostasis in various tissues, including the liver, adipose tissue, and colon [62]. Therefore, *Clps* expression would be a potential early marker of the development of obesity, insulin resistance, and/or colon cancer.

## Conclusions

In conclusion, our data indicate that a few genes primarily involved in lipid metabolism play a functional role in diet-induced pathological changes in the colon. Genome-wide analyses performed in the present study provide new insights on selecting novel genes that may be associated with the development of diseases of the colon. Further studies assessing the functions of these selected genes are necessary to verify them as novel biomarkers for the prevention, early detection, and treatment of obesity-induced CRC.

## Additional files

Additional file 1: Table S1. Composition of the experimental diets.
Additional file 2: Table S2. Food intake and food efficiency ratio of C57BL/6N mice fed normal or high-fat diet for 12 weeks. Data are expressed as mean ± SEM (*n* = 10/group). Asterisks indicate significant differences between mice in the two diet groups according to Student’s *t* test (**P* < 0.001).
Additional file 3: Figure S1. Two-dimensional hierarchical clustering analysis of fold changes in gene expression during the development of diet-induced obesity in **a** normal diet- and **b** high-fat diet-fed C57BL/6N mice. A color gradient from *green to red* indicates low- and high-fold change, respectively.
Additional file 4: Table S3. Genes affected by high-fat diet at different time points in the colon tissue of C57BL/6N mice. Log_2_ fold change of ≥1 or ≤−1 corresponds to a fold change of ≥2 or ≤−2, respectively, based on a comparison between high-fat diet- and normal diet-fed mice at each time point (*P* < 0.05).
Additional file 5: Table S4. Biological processes associated with high-fat diet-responsive genes at different time points in the colon tissue of C57BL/6N mice.

## Abbreviations

*Apoa 4*: Apolipoprotein A-IV
*Cel*: Carboxyl ester lipase
*Clps*: Colipase, pancreatic
CRC: Colorectal cancer
HFD: High-fat diet
IBD: Irritable bowel syndrome
ND: Normal diet
*Ppap2b*: Phosphatidic acid phosphatase type 2B
qPCR: Quantitative polymerase chain reaction
