# Supplementary material for: Time-course microarray analysis for identifying candidate genes involved in obesity-associated pathological changes in the mouse colon
Source: Genes Nutr. 2016 Nov 22;11:30. doi: 10.1186/s12263-016-0547-x (PMC5120484; doi:10.1186/s12263-016-0547-x)
Supplement: Additional file 1: Table S1. — Composition of the experimental diets. [file 12263_2016_547_MOESM1_ESM.docx]

**Table S1. Composition of the experimental diets.**

| **Diet component** | **Normal diet** | **High-fat diet** |
| --- | --- | --- |
| Corn starch | 150 | 111 |
| Sucrose | 500 | 370 |
| Cellulose powder | 50 | 50 |
| Casein | 200 | 200 |
| D,L-methionine | 3 | 3 |
| Corn oil | 50 | 30 |
| Lard | 0 | 170 |
| Cholesterol | 0 | 10 |
| Mineral mixture | 35 | 42 |
| Vitamin mixture | 10 | 12 |
| Choline bitartrate | 2 | 2 |
| tetra-Buthyhydroquinone | 0.01 | 0.04 |
| Lipid, % Energy | 11.65 | 40.86 |
| Total energy, kcal/kg | 3862.00 | 4626.00 |
