# Supplementary material for: Time-course microarray analysis for identifying candidate genes involved in obesity-associated pathological changes in the mouse colon
Source: Genes Nutr. 2016 Nov 22;11:30. doi: 10.1186/s12263-016-0547-x (PMC5120484; doi:10.1186/s12263-016-0547-x)
Supplement: Additional file 2: Table S2. — Food intake and food efficiency ratio of C57BL/6N mice fed normal or high-fat diet for 12 weeks. Data are expressed as mean ± SEM (n = 10/group). Asterisks indicate significant differences between mice in the two diet groups according to Student’s t test (*P < 0.001). [file 12263_2016_547_MOESM2_ESM.docx]

**Table S2. Food intake and food efficiency ratio of C57BL/6N mice fed normal or high-fat diet for 12 weeks.**

|  | **Week** | **Normal diet** | | | **High-fat diet** | | |
| --- | --- | --- | --- | --- | --- | --- | --- |
| Food intake (g/day) | 2 | 2.92 | ± | 0.02 | 2.72 | ± | 0.02 |
|  | 4 | 2.91 | ± | 0.03 | 2.65 | ± | 0.04 |
|  | 8 | 3.36 | ± | 0.03 | 2.68 | ± | 0.03 |
|  | 12 | 3.37 | ± | 0.03 | 2.75 | ± | 0.05 |
| Food efficiency ratio | 2 | 0.08 | ± | 0.002 | 0.12 | ± | 0.003^*^ |
|  | 4 | 0.08 | ± | 0.002 | 0.12 | ± | 0.005^*^ |
|  | 8 | 0.06 | ± | 0.002 | 0.11 | ± | 0.003^*^ |
|  | 12 | 0.06 | ± | 0.001 | 0.10 | ± | 0.002^*^ |

Data are expressed as mean ± SEM (*n* = 10/group). Asterisks indicate significant differences between mice in the two diet groups according to Student's *t-*test (**P* < 0.001).
