# Supplementary material for: Time-course microarray analysis for identifying candidate genes involved in obesity-associated pathological changes in the mouse colon
Source: Genes Nutr. 2016 Nov 22;11:30. doi: 10.1186/s12263-016-0547-x (PMC5120484; doi:10.1186/s12263-016-0547-x)
Supplement: Additional file 3: Figure S1. — Two-dimensional hierarchical clustering analysis of fold changes in gene expression during the development of diet-induced obesity in a normal diet- and b high-fat diet-fed C57BL/6N mice. A color gradient from green to red indicates low- and high-fold change, respectively. [file 12263_2016_547_MOESM3_ESM.pptx]

## Slide 1
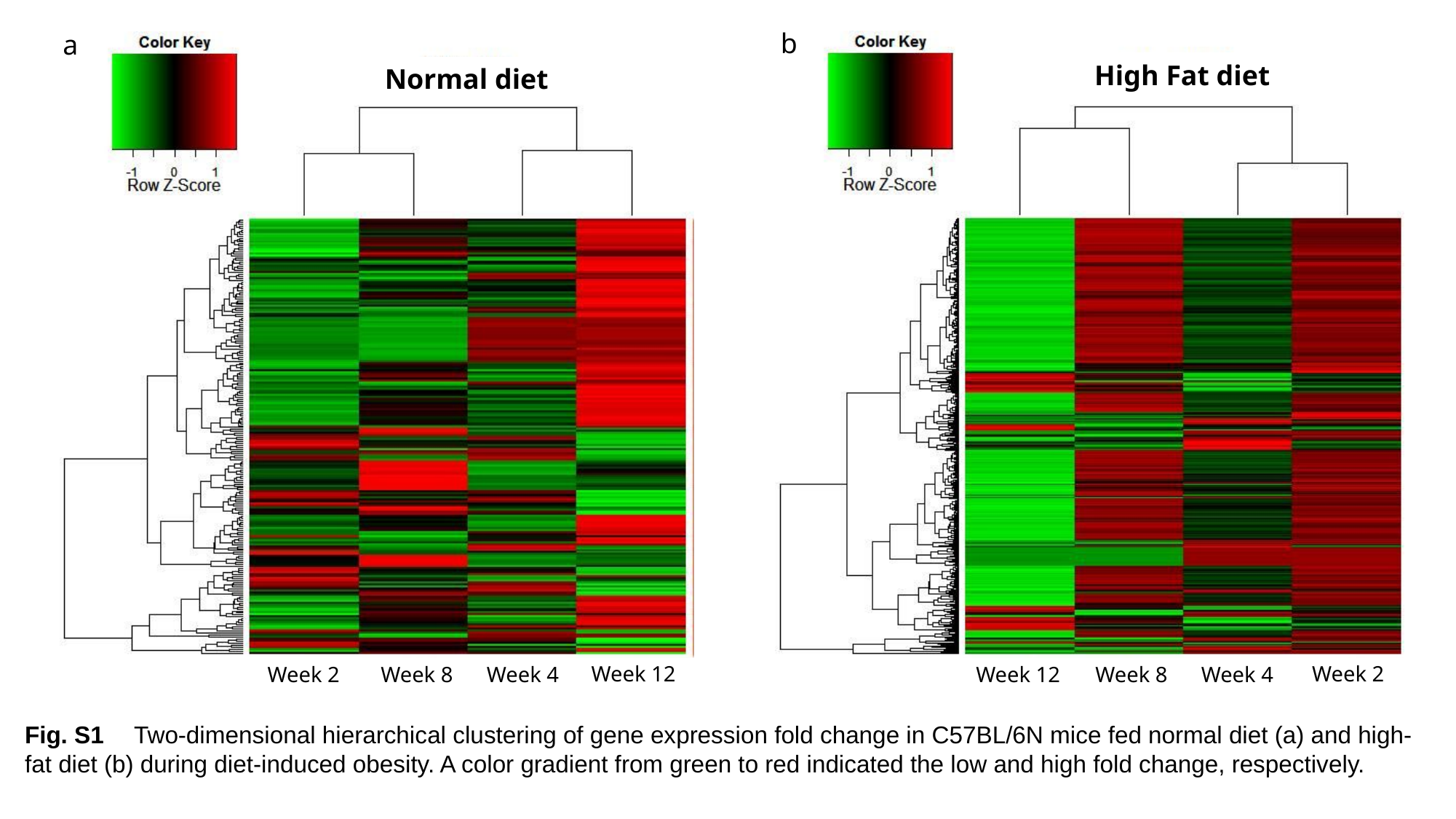

b
High Fat diet
Week 2
Week 8
Week 4
Week 12
a
Normal diet
Week 12
Week 8
Week 4
Week 2
Fig. S1	Two-dimensional hierarchical clustering of gene expression fold change in C57BL/6N mice fed normal diet (a) and high-fat diet (b) during diet-induced obesity. A color gradient from green to red indicated the low and high fold change, respectively.
